# Predicting chromosome damage in astronauts participating in international space station missions

**DOI:** 10.1038/s41598-021-84242-5

**Published:** 2021-03-05

**Authors:** Alan Feiveson, Kerry George, Mark Shavers, Maria Moreno-Villanueva, Ye Zhang, Adriana Babiak-Vazquez, Brian Crucian, Edward Semones, Honglu Wu

**Affiliations:** 1 NASA Johnson Space Center, Houston, TX 77058 USA; 2 KBR, Houston, TX 77058 USA; 3 Human Performance Research Centre, Department of Sport Science, University of Konstanz, Box 30, 78457 Konstanz, Germany; 4 Kennedy Space Center, Cape Canaveral, Florida, USA

**Keywords:** Cancer, Environmental sciences, Health occupations

## Abstract

Space radiation consists of energetic protons and other heavier ions. During the International Space Station program, chromosome aberrations in lymphocytes of astronauts have been analyzed to estimate received biological doses of space radiation. More specifically, pre-flight blood samples were exposed ex vivo to varying doses of gamma rays, while post-flight blood samples were collected shortly and several months after landing. Here, in a study of 43 crew-missions, we investigated whether individual radiosensitivity, as determined by the ex vivo dose–response of the pre-flight chromosome aberration rate (CAR), contributes to the prediction of the post-flight CAR incurred from the radiation exposure during missions. Random-effects Poisson regression was used to estimate subject-specific radiosensitivities from the preflight dose–response data, which were in turn used to predict post-flight CAR and subject-specific relative biological effectiveness (RBEs) between space radiation and gamma radiation. Covariates age, gender were also considered. Results indicate that there is predictive value in background CAR as well as radiosensitivity determined preflight for explaining individual differences in post-flight CAR over and above that which could be explained by BFO dose alone. The in vivo RBE for space radiation was estimated to be approximately 3 relative to the ex vivo dose response to gamma irradiation. In addition, pre-flight radiosensitivity tended to be higher for individuals having a higher background CAR, suggesting that individuals with greater radiosensitivity can be more sensitive to other environmental stressors encountered in daily life. We also noted that both background CAR and radiosensitivity tend to increase with age, although both are highly variable. Finally, we observed no significant difference between the observed CAR shortly after mission and at > 6 months post-mission.

## Introduction

Space radiation consists of high energy protons and heavy charged particles^1^. For spacecraft in low Earth orbits such as the International Space Station (ISS), protons trapped in the van Allen belt, high energy ions from galactic cosmic radiation (GCR) that are not deflected by the geomagnetic field and particles released from solar particle events (SPE) are three of the major sources of radiation. Inside the ISS, secondary particles including neutrons are also generated as the primary particles penetrate through the shielding materials of the spacecraft. High energy charged particles, which possess high linear energy transfer (LET) are known to cause more damage in comparison to gamma or X-rays^1^, Health risks from exposure to cosmic radiation have been a major concern for astronauts since the early days of the manned space program^2^. Exposure to space radiation may result in cancer and other health consequences, and can potentially impact the success of future exploration missions to Mars^3^. Assessing the health risks associated with space-radiation is challenging due the complexity of the space radiation environment and other confounding spaceflight-associated factors such as microgravity and confinement^4–6^.

One measurable effect of exposure to space radiation is an increase in chromosome aberrations (CA) in astronauts’ peripheral blood lymphocytes. CA yields are a reliable biodosimetry tool for estimating an individual’s radiation dose after accidental exposure scenarios^7,8^. NASA’s analysis of chromosome aberrations in blood samples collected from crewmembers before and after space missions started when US astronauts flew on the Russian Mir Station for extended periods^9,10^. After a 9-day Space Shuttle mission, biodosimetry analyses were performed on 2 crewmembers; however no change in chromosome aberration frequency was detected in this short-duration mission^9,11^. Since the beginning of the International Space Station (ISS) program, biodosimetry analysis has also been performed on US ISS astronauts^12,13^. Increases in CA in the blood lymphocytes from Russian cosmonauts and other European astronauts have also been reported^14–16^. Recent efforts undertaken by other space agencies for conducting similar studies are also underway^17^. Although molecular markers, such as gene expression or protein induction have been used to reconstruct radiation doses in humans, chromosomal damage assessed in lymphocytes remains the most reliable biomarker for biodosimetry purposes^18,19^.

In this paper, we present an analysis of CA data collected from astronauts` blood cells before and after ISS missions. Blood samples were obtained from 38 astronauts who participated in ISS missions from 2001 to 2013. Durations of these missions ranged between 2 and 7 months. Five of these astronauts participated in two space missions, thus creating the opportunity to investigate the effect of repeat missions and at the same time to increase the number of crew-missions from 38 to a total of 43. Two main data analyses were performed. The first analysis was intended to assess the degree to which prediction of post-flight chromosome aberration rates could be improved by incorporating information from subject-specific ex vivo pre-flight dose–response models as opposed to prediction based exclusively on BFO doses. In the second analysis, we estimated subject-specific dose-equivalent ratios (analogous to the RBE) by comparing the preflight effect of ex vivo laboratory gamma irradiation with the in vivo effect of real space radiation as manifested in post-flight data. In addition, spin-off analyses were conducted in order to investigate post-flight recovery patterns as well as the effects of mission duration, repeat ISS missions, and the solar cycle. All data analyses were performed using a random-effects version of Poisson regression with age and gender as covariates (*Statistical Methods*).

## Materials and methods

### Subjects and samples

Blood samples were collected from 38 crew members participating in long-duration ISS missions. Because five of these crewmembers participated in two separate missions (ranging from 3 to 9 years apart), a total of 43 crew-missions was potentially available for data analysis. The age of these crewmembers was between 37 and 57 at the time of their ISS mission, and 10 of the crew-members were female. The shortest duration of the ISS missions studied was 67 days and the longest 215 days, resulting in an average BFO dose of 0.028 Gy. For each of the crewmembers, samples were initially collected 1 month before launch. Immediately after blood draw, samples were exposed to an acute dose of 0.1, 0.2, 0.3, 0.4, 0.8 or 2 Gy of gamma rays at the NASA Johnson Space Center at a high dose rate. Since the average dose received during the ISS missions is 28 mGy, gamma-ray dose points above 0.5 Gy were not used in this study. Even though 0.5 Gy is more than 10 times higher than the dose received in space, it is necessary to retain this dose point to allow enough points (4 in this case) to enable reasonably accurate estimation of subject-specific dose–response models and radiosensitivities (see Statistical Methods).

In order to assess the chromosomal damage due to space flight and compare with pre-flight dose–response outcomes, post-flight blood samples from 39 of the 43 crew-missions were collected between 2 and 4 weeks after landing. Furthermore, in order to investigate long-term effects, blood samples from most crewmembers were additionally collected 6 months (or longer) after landing. Our study follows an IRB protocol approved by the NASA Johnson Space Center Institutional Review Board. All methods were carried in accordance with relevant guidelines and regulations. The informed consent was obtained from all crewmembers participating in this study. Analysis of similar pre-flight and post-flight chromosome aberration data collected from 19 of the ISS crew members studied here has been published previously^12^. Also, pre-flight dose–response CA data has been published for 34 of the crewmembers studied here^13^, but post-flight data for 29 of them has not yet been published.

### Chromosome aberration analysis

Detailed methods for collection of blood samples and chromosome analysis have been reported previously^9^. Briefly, astronauts’ venous blood samples were collected in vacutainer tubes containing sodium heparin, at various times before and after flight. Preflight blood samples were exposed to Cs-137 gamma rays at a high dose rate at NASA Johnson Space Center. Immediately after gamma irradiation, cells were cultured in growth medium and chromosomes were subsequently collected following the standard protocol as described^9^. Post-spaceflight blood samples were cultured in growth medium for chromosome collection without ex vivo gamma irradiation. Chromosomes harvested were within the first cell cycle after stimulation.

Chromosome spreads were hybridized in situ with three fluorescence-labeled chromosome specific painting DNA probes in different colors; red, green, and yellow (i.e. a 1:1 combination of green and red probes that fluoresces yellow under a triple band pass filter set)^12^. In most cases, chromosomes 1, 2, and 4 were analyzed. However, some of the earlier data was obtained using chromosomes 1, 2, and 5. For a direct comparison of data using different probe combinations, the frequencies of exchanges in individual chromosomes were extrapolated to whole genome equivalents^12^. On average around 8000 cells were analyzed from each unirradiated pre-flight and all post-flight blood sample. The numbers of the cells analyzed were fewer in gamma-irradiated samples, for which higher aberration counts were expected. See the Appendix for tables of average number of samples and average aberration counts for both pre- and post-flight samples.

Two bicolor chromosomes each containing a centromere were classified as an apparent reciprocal translocation, and recorded as a single exchange event. Reciprocal translocations were classified as stable type aberrations^12^. A dicentric was identified by one bicolor exchange containing two centromeres and a corresponding fragment with no centromere. We assumed visibly incomplete translocations and dicentrics (or one-way exchanges) contained reciprocal fragments that were below the level of detection (as indicated previously^20^), and therefore pooled this data with complete exchanges. Complex exchanges were scored when it was determined that an exchange involved a minimum of three breaks in two or more chromosomes. Total exchanges were calculated by adding the number of apparently simple translocations, dicentrics, incomplete translocations, incomplete dicentrics, and complex exchanges. All data were screened carefully for clonal exchanges. If a clone was identified it was counted as a single exchange^12^. Here, we report results from data analysis of total exchanges, however we also made a parallel analysis of stable translocations (see Appendix).

### Physical dosimetry and BFO dose and BFO dose equivalent calculation

Various radiation dosimeters were used on the ISS to assess the radiation environment and to measure the dose received by the crewmembers, including tissue equivalent proportional counters (TEPC) and crew passive dosimeters (CPD)^21^. Because a CPD measures the dose received at the surface of the crewmember´s chest, the degree of exposure to internal organs was estimated using the computer codes that calculate the dose by modeling the transporting of particles in the environment to the organ of interest^22^. These codes include BRYTRN and HZETRN for trapped protons and GCR particles, respectively^22^. Standard male and female sizes of astronauts were first assumed when computing the dose, which was then normalized to the CPD measurement for individual crewmembers. The body shield distribution for individual organs was provided by computerized anatomical models representing the 50th percentile US Air Force male and female^23,24^. In the model, 32 locations throughout the body are selected to represent the BFO, and the overall body shielding distribution is derived from the average of the 32 locations^23,24^. It has been estimated that the errors in organ dose estimates are about 15%^25^.

### Statistical methods

For each blood sample, let *k* be the number of chromosome aberrations (either total exchanges or stable translocations) observed in *n* lymphocytes. Under assumptions that a) aberrations occur independently, b) *λ*, the probability of an aberration in a given cell is small while the number of cells sampled is large, and c) the probability of more than one aberration in a cell is negligible, *k* is approximately Poisson distributed with mean *nλ.* The Poisson approximation is appropriate here where observed CARs are less than 0.04 and *n* ranges from 88 to over 16,000. In Poisson regression, log *λ* is represented as a function of explanatory variables (see below). For a discussion of Poisson regression for dose–response modeling, see^26^. Separate Poisson regression models were fitted with a) pre-flight data only (excluding the baseline observations) b) post-flight plus baseline data, and c) all data, pre- and post-flight.

#### Pre-flight model

Let ${x}_{ij}$ be the gamma-ray dose in Gy for the *j*-th exposure of cells from the *i*-th subject, and let ${k}_{ij}$ be the corresponding observed total aberration count in ${n}_{ij}$ cells. Then we used the following random-effects version of a Poisson regression model for ${k}_{ij}$:

1 $${k}_{ij}\sim Poisson\left({n}_{ij}{\lambda }_{ij}\right)$$

2 $$\mathrm{log} {\lambda }_{ij}={\beta }_{0}+{{W}_{0i}+(\beta }_{1}+{W}_{1i}){x}_{ij}+{\beta }_{2}{A50}_{i}+{\beta }_{3}{F}_{i}$$

where ${A50}_{i}$ is the subject’s age centered at 50 years, and ${F}_{i}$ is an indicator variable for gender (0 = male, 1 = female). The random effects ${W}_{0i}$ and ${W}_{1i}$ are subject-specific perturbations to the intercept (${\beta }_{0}$) and slope with respect to dose (${\beta }_{1})$. For purposes of describing this model and the post-flight and RBE models (see below), “subject” or “crewmember” refers to each of the 43 crew-mission participants. When referring to actual individuals, some of whom participated in two missions, we will use the term “astronaut”. In this model, it is assumed that ${W}_{0i}$ and ${W}_{1i}$ $\left(i=1, 2. .,43\right)$ follow a bivariate normal distribution. Incorporation of random effects into the model are necessary because it cannot be assumed that the parameters ${\beta }_{0}$ and ${\beta }_{1}$ of the dose response model are the same for subjects with varying sensitivities. This model was fitted in Stata Statistical Software^27^ by maximizing the unconditional Poisson likelihood. Standard errors of all parameter estimates were obtained from the inverse of the information matrix obtained after likelihood maximization. For further study of radiosensitivity as a function of age, we calculated ${B}_{i}$ the average slope of the fitted dose response function for the *i*-th individual by

3 $${B}_{i}=\frac{{\widehat{\lambda }}_{iJ}-{\widehat{\lambda }}_{i0}}{{x}_{iJ}-{x}_{i0}}$$

where ${\widehat{\lambda }}_{ij}$ is the predicted value of ${\lambda }_{ij}$ after fitting the model (Eq. 2), where J is the value of j corresponding to the maximum dose ${x}_{iJ}$ and j = 0 denotes the baseline dose (${x}_{i0}$ = 0). Note that because of nonlinearity ${B}_{i}$ is only approximately equal to the predicted value of ${\beta }_{1}+{w}_{1i}$. First, we fit this model with all pre-flight observations to obtain the best possible characterization of the ex vivo dose–response model; however in order to preserve independence of results between pre- and post-flight analyses, baseline observations (${x}_{ij}=0$) were excluded from a second fitting of this model and were instead used in the post-flight model (see Eq. 4). In addition, ${w}_{0i}$ and ${w}_{1i}$, predicted values of ${W}_{0i}$ and ${W}_{1i}$ respectively were obtained as empirical-Bayes posterior means^28,29^ for later use in the post-flight model.

#### Post-flight model

The post-flight Poisson regression model resembled Eqs. (1) and (2) in form, but with the following differences:

- ${k}_{i0}$ denotes the baseline (pre-flight, no irradiation) aberration count for the i-the subject
- ${k}_{ij}$ denotes the aberration count for the j-th post-mission sample of cells from the i-th subject (*j* = 1, 2, . . .)
- For *j* > 0, ${x}_{ij}={BFO}_{i}$, where ${BFO}_{i}$ is the BFO mission dose received by the i-th subject.
- For *j* = 0, there is no irradiation, so ${x}_{i0}=0.$
- ${w}_{0i}$ and ${w}_{1i}{x}_{ij}$ are included as additional explanatory variables along with age and gender.

With the incorporation of the above, Eq. (2) is replaced by Eq. (3) for the post-flight prediction model:

4 $${\mathrm{log }\lambda }_{ij}={\beta }_{0}+{{U}_{0i}+\beta }_{1}{x}_{ij}+{\beta }_{2}{A50}_{i}+{\beta }_{3}{F}_{i}+{\beta }_{4}{w}_{0i}+{\beta }_{5}{w}_{1i}{x}_{ij}$$

In this model, ${U}_{0i}$, is an error term representing post-flight subject-specific perturbation to the intercept not explained by other terms in the model. Here, ${\beta }_{5}{w}_{1i}$ can be interpreted as the degree to which ${\beta }_{1}$, the post-flight sensitivity to BFO dose is modified by subject-specific perturbations to sensitivity (${w}_{1i}$) to gamma radiation in the preflight ex-vivo series of exposures.

### RBE estimation model

For purposes of estimating subject-specific and average RBE, all data is used in one analysis. More specifically, ${k}_{ij}$ now denotes the aberration count for the *j*-th sample of cells from the i-th subject, including all pre-and post-flight samples. In this model, the index *j* runs from 1 to ${J}_{i}$, where ${J}_{i}$ is the total number samples obtained from the *i*-th subject (both pre- and post-flight).

The model now becomes

5 $$\mathrm{log} {\lambda }_{ij}={\beta }_{0}+{{U}_{0i}+(\beta }_{1,pre}+{U}_{1i}){x}_{pre,ij}+{\beta }_{1,post}{x}_{post,ij}+ {\beta }_{2}{A50}_{i}+{\beta }_{3}{F}_{i},$$

where ${x}_{pre,ij}$ is the gamma-radiation dose applied to the j-th sample of cells if that sample was obtained pre-flight; otherwise ${x}_{pre,ij}$= 0. Conversely, ${x}_{post,ij}={B}_{i}$ if the sample is post-flight; otherwise ${x}_{post,ij}$= 0. The terms ${U}_{0i}$ and ${U}_{1i}$ are random perturbations to ${\beta }_{0}$ and ${\beta }_{1,pre}$,as in the pre-flight model.

Let ${x}^{*}$ be the amount of gamma radiation needed to produce the same expected aberration count as an inflight BFO dose *x*. Then the RBE is defined as the ratio ${x}^{*}/x$. From Eq. (4), it can be seen that for the i-th subject ${({\beta }_{1,pre}+{U}_{1i}) x}^{*}={\beta }_{1,post}x$, thus the RBE for the subject would be

6 $${RBE}_{i}=\frac{{\beta }_{1,post}}{({\beta }_{1,pre}+{U}_{1i})}$$

For an average subject ($${U}_{1i}=0)$$, the RBE is simply

7 $$RBE={\beta }_{1,post}/{\beta }_{1,pre}$$

*Note*: There was not enough range of post-flight dose values to estimate subject-specific perturbations ${U}_{2i}$ to ${\beta }_{1,post}$. If values of ${U}_{2i}$ were available, the subject-specific RBE-values would be $\frac{{\beta }_{1,post}+{U}_{2i}}{({\beta }_{1,pre}+{U}_{1i})}$ instead of Eq. (5). Thus, it is likely that the range of RBE values given by Eq. (5) is conservative because their estimated variation is reduced by not including ${U}_{2i}$.

### Repeat fliers

Five of the 38 astronauts in this study participated in two missions. To investigate whether the pre-flight baseline or dose–response sensitivity for those astronauts had changed between missions we fit an additional pre-flight mixed-effects Poisson regression model with age and mission number included as an explanatory variables. The inclusion of age as a predictor accounts for age differences between missions, thus allowing unbiased estimation of the mission effect on baseline and/or dose sensitivity This model estimated random effects for each of the 38 distinct astronauts as opposed to the previous models that estimated random effects for each of the 43 total crew-missions.

## Results

### Chromosome aberration rates (CARs)

**Figure 1:**
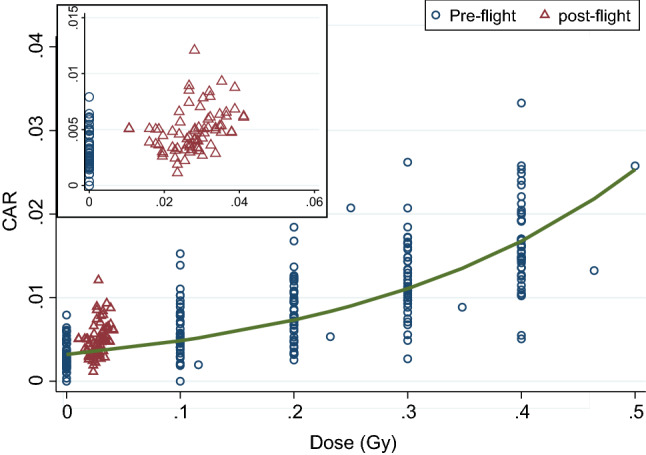
Observed chromosome aberration rate (CAR) in lymphocytes collected from the ISS crewmembers pre-flight (blue circles) and post-flight (red triangles), plotted against dose where "dose" is either the *ex-vivo* gamma-ray dose in Gy (pre-flight) or the estimated BFO dose in Gy, received during flight. The solid curve shows the mean dose–response function estimated from Poisson regression on the pre-flight samples. The average BFO dose received during ISS missions was 0.028 Gy, and the average CAR post-mission was 0.0049 (insert), reflecting an average RBE of about 3.1 (see *RBE Estimation Model)*. Results presented in this and other figures were generated using Stata Statistical Software^27^.

Pre-flight observations at doses greater than 0.5 Gy were discarded because we were primarily interested in the low-dose response. For all remaining observations (both pre- and post-flight), Fig. 1 shows the CAR for total aberrations plotted against radiation dose, where "Dose" is the gamma- ray dose in Gy (preflight samples) or the estimated BFO dose in Gy as received during flight (post-flight samples). The tendency of the post-flight observations of CAR to lie above the pre-flight dose response line is reflecting an RBE of about 3.1.

### Pre-flight dose–response model

**Figure 2:**
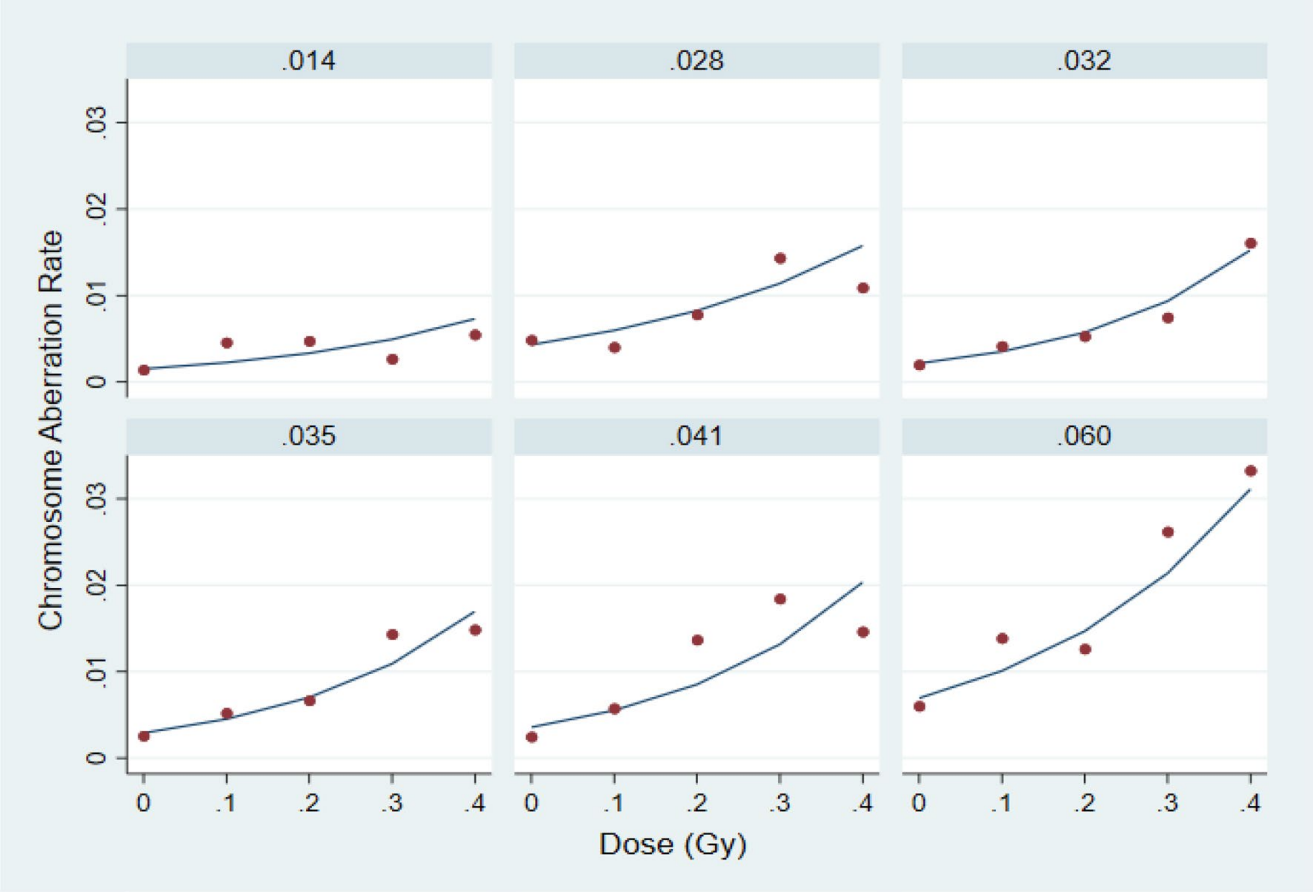
Poisson regression predictions of pre-flight dose response (solid lines) for 6 crewmembers covering the observed range of pre-flight average slopes (values at the top of each section are in the units of CAR per Gy of dose). Observed CARs are plotted as red circles.

After discarding high-dose data, the mixed-effects Poisson regression model (Eqs. 1–2) was applied to all remaining pre-flight observations, resulting in individual dose–response models reflecting differences in ${W}_{0i}$ and ${W}_{1i}$, as well as age and gender. Examples of these subject-specific dose–response models are shown in Fig. 2 for 6 subjects whose average slopes varied between 0.014 (minimum) and 0.060 (maximum). Parameter estimates and standard errors for the overall mixed-effects model are shown in Table 1. This table shows that the predicted baseline CAR for a male subject at age 50, would be *exp*(− 5.72) = 0.0033; however, for some subjects this value could be expected to be as high as exp(− 5.72 + 0.46) = 0.0052 or as low as exp(− 5.72 − 0.46) = 0.0021 where the value 0.46 is the estimated SD of ${W}_{0i}$. Also, for an average subject, we would expect the CAR to increase by a factor of *exp*(4.15/10) = 1.51 (51%) for each 0.1 Gy of gamma radiation exposure, however for particular subjects this factor could be as high as exp((4.15 + 0.88)/10) = 1.65 (65%), or as low as exp((4.15 − 0.88)/10) = 1.39 (39%), where the increment 0.88 is 1 SD of ${W}_{1i}$. In addition, we estimated that on average, baseline CAR would increase by a factor of exp(0.029) = 1.03 (2.9%) for each year of age. The effect of age was also manifested in the radiosensitivies ${B}_{i}$, obtained from Eq. (3), which were weakly correlated with age (r = 0.34; 95% conf = (+ 0.05, + 0.58). Similarly, estimates of background CAR ($\mathrm{exp}({\beta }_{0}+{W}_{0i})$ also appeared weakly correlated with age (r = 0.24, 95% conf (− 0.06, + 0.50). See Figures A1 and A2 (Appendix) for illustrations of these effects. For an average female subject (${W}_{oi}=0$) at age 50 years, the baseline CAR was estimated to be about exp(− 5.717 + 0.168) = 0.0038 as opposed to exp(− 5.717) = 0.0033 for an average male subject. However, there was not enough evidence in support of an actual gender effect (*p* = 0.15).

**Table 1:** Pre-flight Ex Vivo poisson regression parameter estimates (“Est.”) and standard errors (“SE”) based on all pre-flight observations with dose ≤ 0.5 Gy. The “Z”-value is the signal-to-noise ratio (Est/SE), while “p” is the *p* value for the test of the null hypothesis that the parameter is zero. In the fitted model, mean log CAR increases by about 0.0415 (4.151/100) per cGy of gamma radiation, corresponding to about a 4% increase in median CAR per cGy of increased radiation. Similarly, all other things being equal, median background CAR increases by about 3% per year of age. There could also be a gender effect, but there were not enough female subjects to accurately estimate it. Results presented in this and the other tables were generated using Stata Statistical Software^27^.

Pre-flight model parameter	Equation 2 predictor variable	Est	SE	Z-value	*p*
β_1_	*x*_*ij*_* (γ-ray dose)*	4.151	0.168	24.72	0
β_2_	*A50*_*i*_	0.029	0.012	2.42	0.016
β_3_	*F*_*i*_	0.168	0.118	1.43	0.153
β_4_	1	− 5.717	0.084	− 68.2	0

### Post-flight dose–response model

The pre-flight Poisson regression model (Eqs. 1–2) was refitted, this time without observations with ${x}_{ij}$= 0, to obtain predicted values (${w}_{0i}, {w}_{1i}$) of the random effects ${W}_{0i}$ and ${W}_{1i}$, which were used in turn as predictors themselves in the post-flight model (Eq. 4). In fitting the post-flight model the pre-flight observations with ${x}_{ij}$= 0 as well as all post-flight observations were used. After re-fitting the pre-flight model, we obtained values of ${w}_{0i}$ and ${w}_{1i}$ for each of the 43 subjects. By definition, means of the random effects are zero; standard deviations and the ranges of ${w}_{0i}$ and ${w}_{1i}$ are given in Table 2.

**Table 2:** Descriptive statistics for predicted random effects (no baseline obs.)

Random effect	Obs	SD	Min	Max
w_0i_ = pred(W0i)	43	.472	− .839	1.077
w_1i_ = pred(W1i)	43	.771	− 1.303	1.973

**Figure 3:**
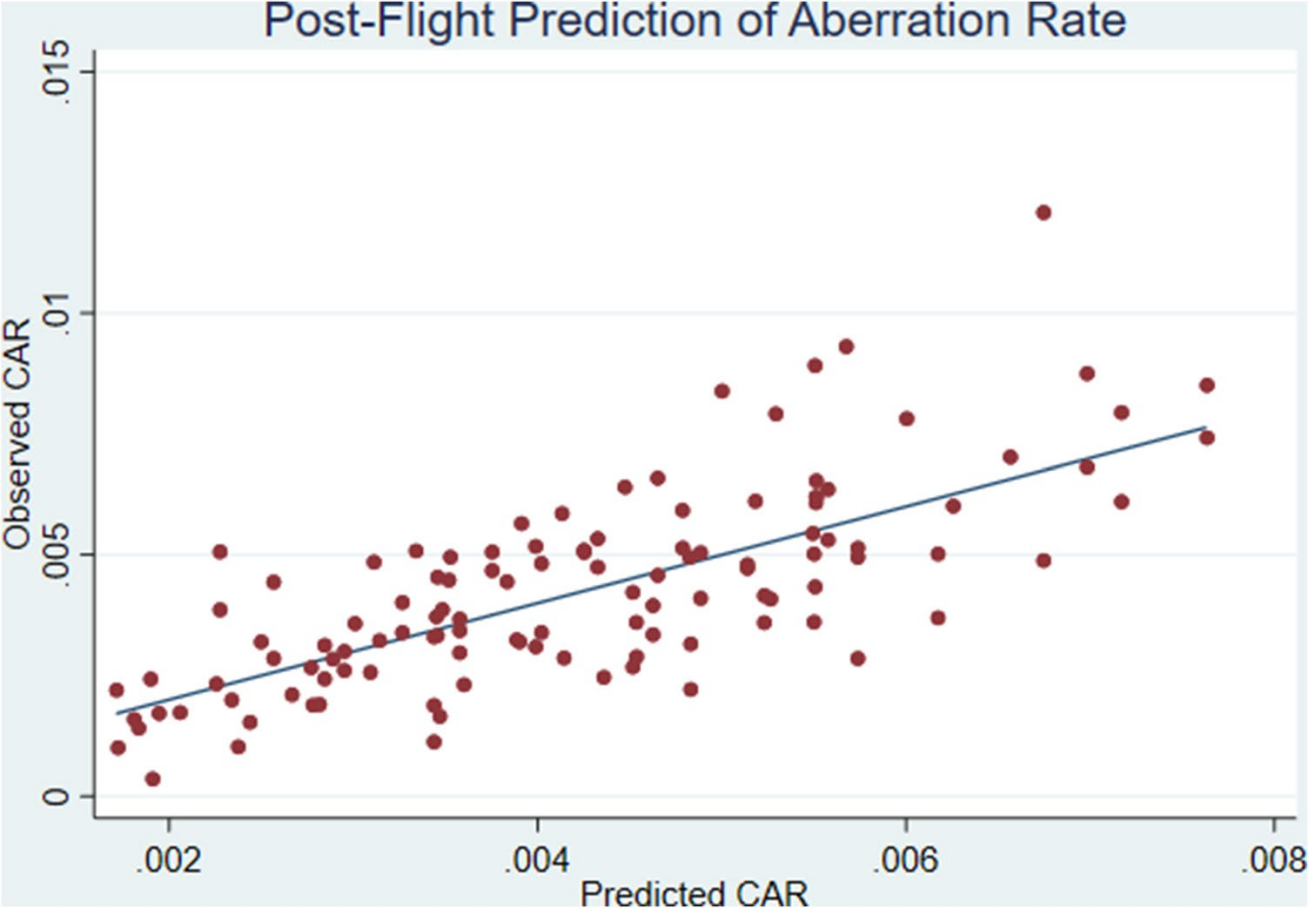
Post-flight prediction of chromosomal aberration rates (CAR). Subject-specific random intercepts (${w}_{0i}$) and sensitivities (${w}_{1i}$) obtained from the pre-flight model were used in this prediction model given by Eq. (4).

After fitting the post-flight model (Eqs. 1, 4) we obtained the parameter estimates in Table 3. As shown in Table 3, the post-flight CAR for an average subject would be about 16% higher (exp(14.746/100) = 1.16) for every increase in BFO dose of 0.01 Gy. However the CAR for subjects with “large” *w*_*1i*_ (*e.g. w*_*1i*_ = 1.5) would increase by 27% (exp((14.746 + 6.268 × 1.5)/100) = 1.27 ) for each increase of 0.01 Gy BFO dose. The significance of the term “w_1_x” in Table 3 (coeff. est. = 6.268, SE = 1.611, *p* < 0.001) is that pre-flight in vivo sensitivity to gamma radiation (manifested through *w*_*1i*_ × x_ij_) also contributes to the prediction of post-flight CAR over and above BFO dose alone. Figure 3 shows actual vs. predicted values of post-flight CAR’s, where the predicted values were obtained using the model parameters in Table 3. Not surprisingly, individual baseline differences, reflected in *w*_*0i*_, were also predictive of the post-flight CAR (coeff. est. = 0.704, SE = 0.087, *p* < 0.001).

**Figure 4:**
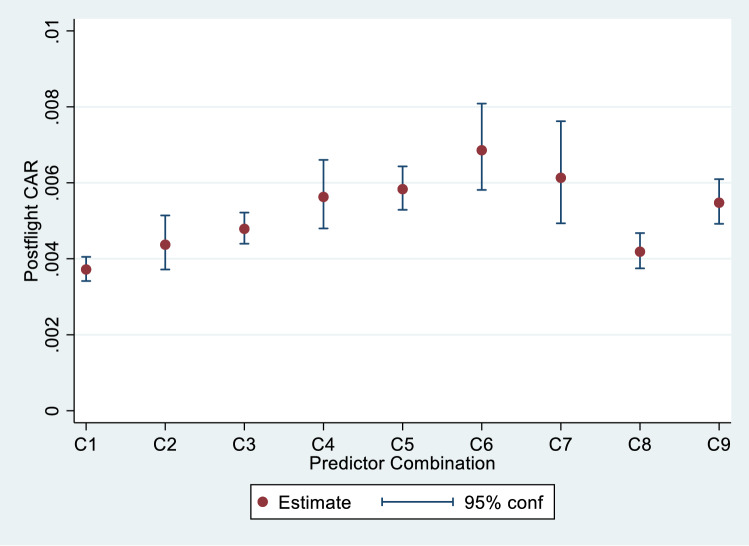
Predicted CAR and 95% confidence limits for 9 predictor combinations.

Figure 4 illustrates the sensitivity of the model and its uncertainty by plotting point estimates of post-flight CAR along with 95% confidence intervals for 9 particular combinations of BFO dose, age, gender, and *w*_*1i*_ (Table 4). These combinations were chosen to encompass the range of observed values of these variables.

**Table 3:** Post-flight in-vivo poisson regression parameter estimates (“Est.”) and standard errors (“SE”) based on all post-flight observations with BFO doses ranging from 0.01 to 0.04 Gy as well as baseline pre-flight observations. Besides age and gender, predictors in this model included predicted values of baseline random subject-specific perturbations to CAR (w_0i_) as well as predicted values of subject-specific perturbations to radiosensitivity (w_1i_x_ij_).

Post-flight model parameter	Equation 4 predictor variable	Est	SE	Z-value	*p*
β_1_	*x*_*ij*_ (*BFO* dose)	14.746	1.202	12.27	< 0.001
β_2_	*A50*_*i*_	0.025	0.009	2.83	0.005
β_3_	*F*_*i*_	0.162	0.083	1.94	0.052
β_4_	w_0i_	0.704	0.087	8.13	< 0.001
β_5_	w_1i_x_ij_	6.268	1.611	3.89	< 0.001
β_0_	_cons	− 5.751	0.048	− 119.08	< 0.001

**Table 4:** Combinations of predictors used in Fig. 4 and model-based estimates of % CAR. For ease of readability, CAR is expressed here as a percent (100 × CAR).

Combination	BFO dose	Age (years)	Female	W_1i_	CAR (%)
C1	0.011	50	0	0.00	0.37
C2	0.011	50	1	0.00	0.44
C3	0.028	50	0	0.00	0.48
C4	0.028	50	1	0.00	0.56
C5	0.041	50	0	0.00	0.58
C6	0.041	50	1	0.00	0.69
C7	0.028	60	0	0.00	0.61
C8	0.028	50	0	− 0.77	0.42
C9	0.028	50	0	0.77	0.55

This figure suggests that the largest source of uncertainty in the prediction of CAR from the post-flight model is the relatively unclear effect of age (combination C7) when relatively distant from the mean of 46.9 years. Combination C7 is the same as C3 (medium dose rate, male, with random effects *w*_*0i*_ and *w*_*1i*_ at their means of zero), except that age = 60 years in C7 and 50 years in C3. The effect of female vs male gender can also be seen comparing C2 with C1, C4 with C3, and C6 with C5. The larger confidence regions for combinations with female indicators (C2, C4, and C6) reflect the small number of females in the study. Finally, comparing C8 and C9 with C1 illustrates the expected effect of preflight radiosensitivity on CAR, where C8 represents a subject with ${w}_{1i}$ = − 0.77 (1 SD below the mean of zero), and where C9 represents a subject with ${w}_{1i}$ =  + 0.77 (1 SD above the mean of zero). The difference in expected CAR between C8 and C9 illustrates how differences in values of ${w}_{1i}$ can contribute to the prediction of CAR over and above BFO dose alone.

### Recovery of CAR after landing

Average observed CAR measured 6–12 months after landing for 31 crew-missions with at least two post-flight time points was not noticeably different than the average CAR on the first post-flight time point measured about 2 weeks after landing (first mean CAR = 0.00490, 2^nd^ mean CAR = 0.00477, *p* = 0.70, paired t-test).

### RBE estimation

**Figure 5:**
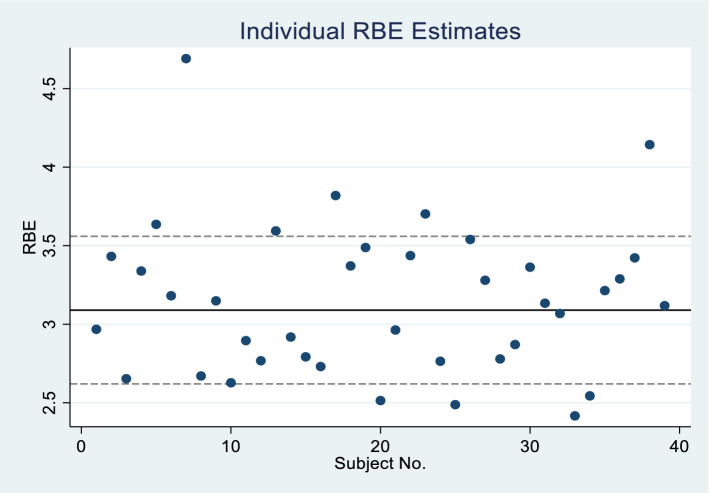
Individual RBE estimates. The RBE for an average subject is represented by the horizontal line with 95% confidence limits (dashed lines). Subject numbers were assigned randomly to preserve sensitive personal information.

After fitting the RBE model (Eqs. 1, 4) to all data (pre- and post-flight), we obtained the parameter estimates as indicated in Table 5. Estimated standard deviations of the random effects ${U}_{0i}$ and ${U}_{1i}$ are in Table 6. Applying Eq. (7) to these estimated results, we calculated that the RBE for a subject with average sensitivity (${U}_{1i}=0$) would be 3.09 ± 0.24. Taking into account the different subject sensitivities, we obtained subject-specific values of RBE ranging from 2.4 to 4.7. However, as pointed out earlier, this range is probably an underestimate of the true range of RBE’s. Figure 5 shows values of individual RBE’s plotted against subject number. Values of the estimated parameters for the RBE model are shown in Table 5.

**Table 5:** Estimates of parameters in RBE model (Eq. 5) based on Poisson regression that includes all observed data, both pre- and post-flight with separate terms for sensitivity to gamma radiation (${\beta }_{1,pre}$) and BFO dose (${\beta }_{1,post}$) as well as overall age and gender effects.

RBE model parameter	Equation (5) predictor variable	Est	SE	Z-value	*p*
β_1,pre_	x_pre,ij_	4.031	0.14	28.7	< 0.001
β_1,post_	x_post,ij_	12.475	1.058	11.8	< 0.001
β_2_	A50_i_	0.027	0.011	2.4	0.018
β_3_	F_i_	0.153	0.11	1.4	0.164
β_0_	1	− 5.679	0.064	− 89.3	< 0.001

**Table 6:** Estimated standard deviations of RBE model random effects.

Effect	Estimate
sd(*U*_1i_))	.680
sd(*U*_0i_)	.320

### Multiple missions

**Figure 6:**
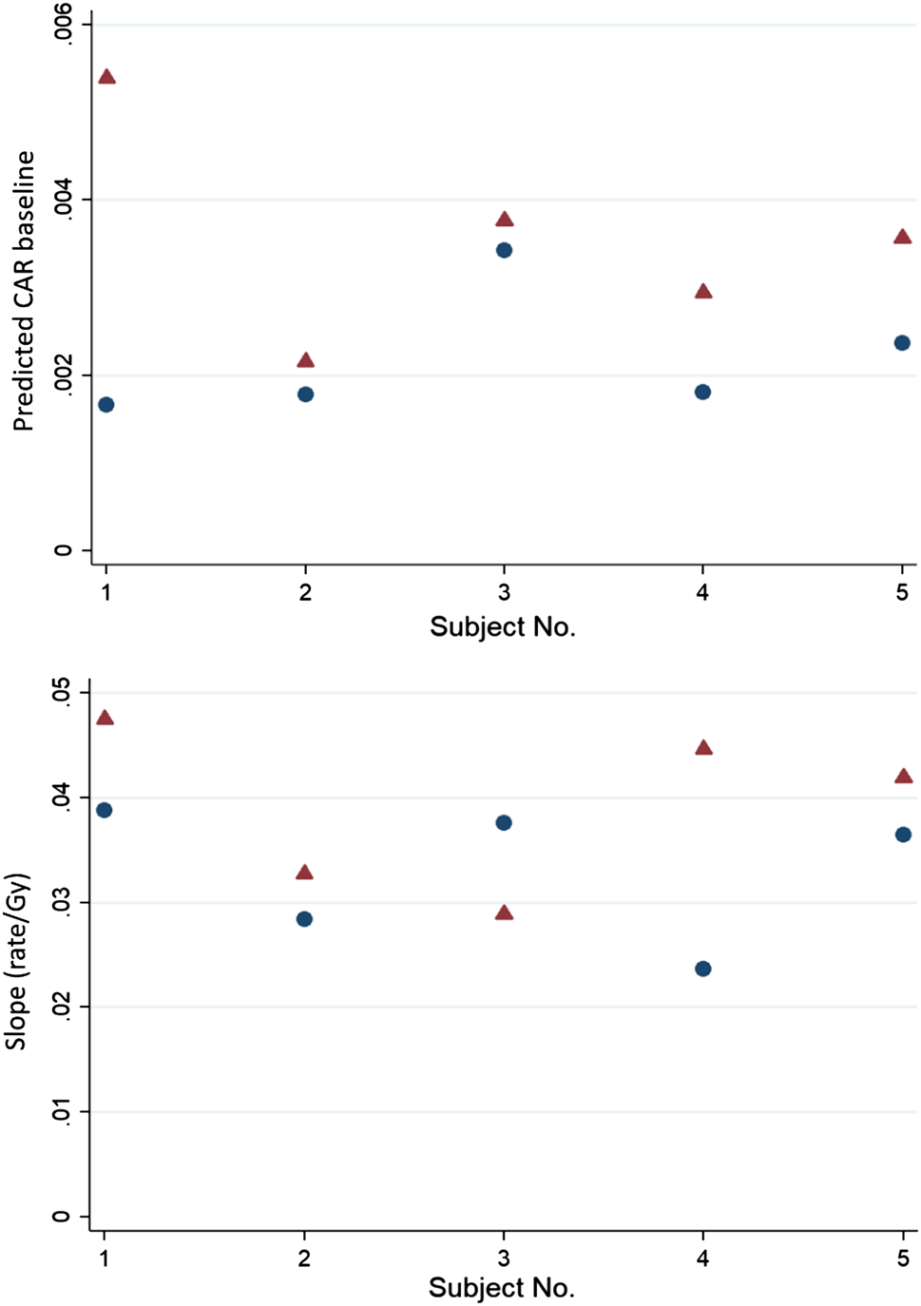
Background CAR (top panel) and average dose–response slope (Eq. 6) (bottom panel) prior to the first (blue circle) and second (red triangle) ISS missions for 5 repeated flyers. The background was about the same or higher prior to the second mission for all 5 of these astronauts, whereas the average slopes were higher prior to the second mission for 4 of these 5 astronauts.

A refitting of the pre-flight Poisson regression model with mission number (1 or 2) and age as explanatory variables for each of the distinct 38 astronauts resulted in an estimated increase of about 50% for the average pre-flight baseline aberration rate for a second mission as compared with a first mission even after adjusting for age increase between missions. However, the small number of multi-mission fliers (5) was reflected in a large uncertainty in the estimated increase; 95% confidence limits = (15%, 92%) (*p* = 0.003). On the other hand, no substantial evidence of a mission effect on the pre-flight dose–response average slope was found; estimated average increase = 23%; 95% confidence limits = (− 8%, + 55%) (*p* = 0.15). Point estimates of intercepts and average slopes are plotted for the 5 astronauts with two missions in Fig. 6.

### Discussion

Both pre- and post-flight chromosome damage was classified in terms total aberrations and stable translocations. Although here we report only the results of analysis of total aberrations, a parallel analysis of stable translocation data showed a similar outcome (see Appendix).

In the biodosimetry study, blood samples collected pre-flight were exposed to gamma rays ex vivo to determine individual dose response curves. After fitting the mixed-effects Poisson regression model (Eq. 2) to the preflight dose–response data, we obtained estimates of the average slope of the dose–response function (log CAR per Gy dose) as well as the background CAR for each subject. Although both of these parameters varied greatly between individuals, they were positively correlated and tended to increase with age (RESULTS—*Pre-Flight Dose–Response Model*).

Radiation is only one of the environmental stressors that causes chromosome damage in the lymphocytes of humans^30^. It has been reported that CA levels can be affected by smoking^31^. Elevated CA are also associated with exposure to toxins ranging from formaldehyde to electronics waste^32,33^. The pre-flight background CAs in the astronauts were partly caused by radiation from prior Space Shuttle and ISS missions, and from aircraft flights and medical procedures. The whole-body dose equivalent received during Space Shuttle missions is typically a fraction of one cSv^12^; however, some of the background CA could have been induced by environmental factors. The positive correlation between the pre-flight slope and background CA suggests that individuals with greater radiosensitivity are also likely to be more susceptible to other environmental stressors.

### Association between ex-vivo dose response and spaceflight-induced CAR

The primary goal of the present study was to determine whether ISS crewmembers´ individual radiosensitivities would contribute to the prediction of their spaceflight-induced CAR over and above BFO dose. A number of methodological strategies for quantifying individual radiosensitivities have been proposed in the past^34,35^. These approaches include the “G2- assay” in which human lymphocytes are stimulated to divide before being exposed to ex vivo radiation, and ex vivo exposure of human resting (G0 phase) lymphocytes to known doses of radiation^36,37^. Using the Poisson regression model, we assessed the degree to which CAR background, and preflight-estimated radiosensitivity were predictive of post-flight CAR in a joint model with BFO dose age and gender. Our analysis revealed that not only did the preflight background and radiosensitivity vary considerably between individuals (Table 2), but that both were found to contribute to the prediction of post-flight CAR over and above what could be explained by BFO dose and demographic factors alone (Table 3). In particular, these results suggest that all other things being equal, individuals with higher radiosensitivities will experience greater chromosomal damage during spaceflight. Similarly, whether radiosensitivity determined from ex vivo exposure predicts in vivo response of tumors to radiotherapy has been widely investigated. A similar regression analysis was performed on a group of healthy retired workers from the British Nuclear Fuels plc facility at Sellafield^38^. It was concluded that only cumulative occupational radiation dose, but neither radiosensitivity nor age, have an influence on chromosome translocation frequencies in vivo. A study on a larger cohort of retired Sellafield workers has also reported no association between polymorphisms in genes involved in the base excision and double strand break repairs and in vivo chromosome aberrations associated with occupational exposure^39^.

### Dose and dose rate effects

The Poisson regression analysis in the present study was performed with the accumulated radiation dose of the BFO dose as an independent variable. The same analysis was also performed using the dose rate as an independent variable, which was calculated by dividing the accumulated BFO dose by the mission duration. Among the crewmembers, the dose rate at the BFO ranged from 0.012 to 0.024 cGy/day. Such a difference is caused primarily by the solar cycle, as the dose rate during solar minimum activity is known to be about twice that during solar maximum activity. Interestingly, the prediction was slightly better when taking the dose rate into account rather than the accumulated dose. Among the crewmembers the dose rate varied depending mostly on the solar cycle and the location of the crewmember inside the ISS. Generally, CAR is higher for higher dose rate, and this is particularly true for low-LET radiation^40^. In low Earth orbits (LEO), the radiation environment is a mixture of protons in the trapped radiation belt, and a fraction of high-LET galactic cosmic radiation (GCR). While high-energy protons produce similar CAR and similar dose-rate dependence as gamma rays, low dose rates of low energy (high-LET) protons and high-LET heavy ions may produce damage that is independent of the dose, particularly taking into account the non-targeted radiation effects. The dependency of dose rate may also be a reflection of the solar cycle, in which the dose rate is greater during solar minimum activities (NCRP 1989). As a result, the fraction of high-LET components may be higher during solar minimum activities, and likely responsible for the dependency of dose rate and post-flight CAR.

### Bone marrow cells vs. circulating peripheral blood mononuclear cells (PBMC)

We observed that on average, CAR detected 2 weeks after landing did not noticeably differ from the CAR detected 6–12 months after landing. In humans, the life span of human lymphocytes varies significantly depending on the subtypes of the cells^41^. In this study, chromosome spreads analyzed for aberrations were mostly from T cells with a small percentage of other cell types^42^. While the estimates of the life span are 164 and 157 days for CD4 + and CD8 + cells, respectively^43^, some of the naïve T cells can take 3 years to divide^44^. The estimated life span may be even longer as suggested by different mathematical models^43^. Chromosome damage analyzed shortly after radiation exposure in vivo represents the damage in mostly circulating T cells. However, if blood is collected years after radiation exposure, as it was in the case of Japanese atomic bomb survivors^45^, chromosome damage reflects the damage induced in the bone marrow precursor cells at the time of radiation. In our study, blood samples were collected at two time points; 2 weeks and again 6–12 months after flight. Taking mission duration into account, the second post-flight collection of blood samples was performed from 12–18 months after launch to the ISS. Based on the present analysis that CAR between and first and second post-flight collections were similar, and that the average life span of T cells is ~ 160 days, we argue that a significant portion of the damage detected in our biodosimetry analysis was a result of the damage caused in bone marrow cells. In fact, chromosome aberrations of clonal origin have been reported in some of the astronauts’ post-flight blood samples^46^.

### Age and gender effects

The present analysis suggests an age effect in the background of chromosome aberration frequencies (Table 1), in the average slope of the dose response in ex vivo gamma exposures, and in post-flight CAR. In general, estimates of age effects had relatively high uncertainty (for example, see Fig. 4.) due to the narrow age range in the ISS astronauts in comparison to the general population. Age dependence in the radiation effects has been reported previously in the literature. Studies of background chromosome aberration rates in healthy adults have indicated that stable aberrations accumulate with time^30^. The dependence of translocation frequencies with age was found to be linear at younger ages, but increases with upward curvature at older ages^30^. Around age 50, the translation frequency increases by 0.24 per 100 cells in 10 years^30^. Increased levels of chromosome structural abnormalities as a function of age have also been shown in spermatozoa^47,48^, an in dermal fibroblasts in individuals^49^. In addition to the background CAR, sensitivities to radiation exposure would be expected to vary with the age of the individual due in part to the decreased efficiency in repairing DNA strand breaks for older individuals^49^. Radiosensitivity was investigated in breast cancer patients and healthy individuals of different age groups by exposing their blood samples ex vivo to radiation, similar to the present study with the pre-flight blood samples^50^. Results of that study showed that the cancer patients were distinctly more radiosensitive compared to healthy controls; but that while radiosensitivity appeared age dependent for the control subjects there was no evidence of a similar relation for the cancer patients. Similarly, a more comprehensive study involving patients with a variety of cancers^51^concluded that radiosensitivity increased with age for healthy patients, but did not appear to change with age in the cancer patients, probably because of a large variation in the patient group. While these two studies^50,51^ used blood samples exposed ex vivo to radiation, analysis of cancer mortality in workers at Oak Ridge National Laboratory indicated that cumulative radiation doses were associated with increases in all-cancer mortality, and that sensitivity to the carcinogenic effects of ionizing radiation may increase with older ages at exposure^52^. The present study shows an increased CAR background as a function of age, which is comparable to the published results.

Gender differences in the effects of space radiation are also a concern for assessing the associated risks^53^. In a study aimed at investigating the effects of smoking on chromosome sensitivity to gamma radiation, human lymphocytes collected from healthy subjects were stimulated to grow before exposure^54^, an approach that differs from the present study where unstimulated lymphocytes were exposed. Results of the previous study^54^ showed that the mean frequency of radiation-induced breaks was significantly higher in men than in women. In the present study, although results suggest a possible differential gender role on some aspects of radiosensitivity, the number of female crewmembers (10) was too small to draw any definite conclusions from the available data. Although it is known that smoking also affects chromosome aberration frequencies^31^, astronauts are mostly non-smokers.

### RBE

In the present analysis, individual RBE estimates were found to range between 2.4 and 4.7 with a mean value of 3.09 ± 0.24 (Fig. 5). These values are similar to those estimated previously^12^. In the ISS orbit, space radiation that the crewmembers are exposed to consists of mostly protons and a small fraction of high-LET heavier ions^1^. Except for the Bragg peak region, RBE values of protons are known to be close to 1 for chromosome damage and for other biological endpoints^55^. The effect of high-LET radiation is traditionally weighted by the quality factor, which is defined to be 1 for low LET, and have a peak value of 20 at LET = 100 keV/μm^56^. Applying the quality factor recommended by the International Commission on Radiological Protection (ICRP 60)^56^ to the radiation environment in the ISS orbit, we estimated the RBE at the depth representing the BFO locations to be about 1.4, which is lower than the average RBE of about 3 that we obtained for in-chromosome aberrations. A number of factors can contribute to this discrepancy. Firstly, the RBE value in the present analysis was derived by comparing the *in-vivo* exposure of a mixture of bone marrow cells and PBMCs to *ex-vivo* exposure of PBMCs. The sensitivities between these bone-marrow cells to *in-vivo* and PBMCs to *ex-vivo* radiation exposure can be different. In addition, *in-vivo* exposure to space radiation occurred under microgravity and other environmental stress conditions associated with the ISS, whereas ex vivo exposure took place on the ground. It has been suggested that the stress factors experienced by the crewmembers in space may affect DNA damage response, resulting in a higher radiosensitivity^6^. Furthermore, the high-LET component of space radiation, even in LEO, may induce the non-target effect^57^. As such, the damage from very low doses of high-LET radiation may be greater than expected^58,59^. As shown in Fig. 1, the RBE in the present analysis was derived based on dose response in the range of BFO dose between 0.01 and 0.04 Gy, which is approximately linear. We argue that the RBE for chronic exposure would be close to the value of 3.1 even though the gamma dose response curve was obtained under the acute exposure scenario. Of course, RBE values for different types of chromosome aberrations can be different, particularly for complex type of aberrations^60^. However, the yield of complex type damage observed in the crewmembers’ samples is so low that RBE values for them could not be determined.

### Repeat fliers

Of the 38 astronauts flown on long-duration ISS mission, 5 participated in two separate long-duration missions. The time between their first and second missions varied from 3 to 9 years^13^. Analysis was carried out to determine whether the first mission affected the radiosensitivity of the crewmembers in the subsequent missions, as determined by changes in the pre-flight dose response in lymphocytes after *ex-vivo* gamma irradiation. As shown in Fig. 6, point estimates of the intercept of the dose–response function were higher for the second missions for all 5 astronauts. Formal analysis taking age and uncertainty into account corroborated a definite mission effect (*p* = 0.002). It is therefore plausible to conclude that the consistent increases are the result of space radiation exposure during the astronauts’ first mission. The average slope of the dose response was higher for the second mission for 4 of the 5 astronauts, but the increase was not statistically significant.

### Limitations

The ability of the post-flight model to predict CAR is limited by errors in the predictors; in particular the estimates of ${W}_{0i}$ and ${W}_{1i}$ were based on limited ex-vivo dose–response data. Of necessity, the control baseline data was withheld in fitting the pre-flight model so that these observations could be used in estimating the post-flight model without duplication. Assumptions of independence and no more than one chromosome aberration per cell may not exactly hold, in which case aberration counts may not have a Poisson distribution. Also, the log-linear form of all our Poisson regression models is at best only an approximation to how the explanatory variables relate to CAR response. Despite these limitations, the root-mean-square error in the post-flight prediction (as illustrated in Fig. 4) is about 0.0015—only slightly higher than the repeatability standard deviation of 0.0013 observed in 73 post-flight observations of CAR. A validation study re-estimating the post-flight model with one subject left out resulted in predictions that were virtually as accurate as the full-sample predictions from Table 3.

### Summary

Our present analysis of biodosimetry data indicates that the background chromosome aberration rate, as well as radiosensitivity as defined by the ex-vivo dose response to gamma irradiation vary considerably among individuals. Radiosensitivities tend to be higher for those having a higher CA background, and both parameters were generally higher in older crewmembers. Post ISS missions, the chromosome aberration rate observed in the crewmembers can be fairly well predicted by the radiosensitivity determined preflight, in combination with the dose received during the mission and the background CAR. Taken together, our analysis suggests that crewmembers with greater radiosensitivity can be more sensitive to not only radiation exposure in space, but also to other environmental stressors experienced prior to the ISS missions. It has been commonly believed that older astronauts would have lower radiation risks, due in part to their shorter remaining life span. However, the present finding that older crewmembers may be more sensitive to space radiation exposure will potentially impact the age dependence in the risk assessment, particularly as humans continuously live longer.

## Supplementary Information

Supplementary Information
