# Supplementary material for: Predicting chromosome damage in astronauts participating in international space station missions
Source: Sci Rep. 2021 Mar 5;11:5293. doi: 10.1038/s41598-021-84242-5 (PMC7935859; doi:10.1038/s41598-021-84242-5)
Supplement: Supplementary file 1 — Supplementary Information [file 41598_2021_84242_MOESM1_ESM.docx]

**Predicting Chromosome Damage in Astronauts Participating in International Space Station Missions**

**APPENDIX**

Alan Feiveson^1^, Kerry George^2^, Mark Shavers^2^, Maria Moreno-Villanueva^1,3^, Ye Zhang^4^, Adriana Babiak-Vazquez^2^, Brian Crucian^1^, Edward Semones^1^ and Honglu Wu^1,*^

^1^NASA Johnson Space Center, Houston, Texas 77058, USA

^2^KBR, Houston, Texas, 77058, USA

^3^Human Performance Research Centre, Department of Sport Science, Box 30, University of Konstanz, 78457 Konstanz, Germany

^4^Kennedy Space Center, Cape Canaveral, Florida, USA

*Corresponding author

Honglu Wu

NASA Johnson Space Center

Houston, Texas 77958

USA

[honglu.wu-1@nasa.gov](mailto:honglu.wu-1@nasa.gov)

**PART ONE – Auxiliary Tables and Figures**

**Table A1. Pre-flight average cells sampled, average aberration counts per subject, by dose range.**

| Pre-Flight Samples | | | |
| --- | --- | --- | --- |
| Dose (Gy) | Average Aberration Count | Average # Cells Sampled per Crew-Mission | # Crew-Missions |
| 0 | 26.4 | 8122.7 | 43 |
| .10 - .12 | 14.9 | 2683.0 | 43 |
| .20 - .25 | 15.7 | 2049.0 | 43 |
| .30 - .35 | 24.1 | 2042.2 | 43 |
| .4 - .5 | 28.9 | 1808.5 | 43 |

**Note: 38 distinct subjects participated in this study, with 5 of them taking part in two missions, making a total of 43 crew-missions studied. Four subjects did not have post-flight chromosome aberration data.**

**Table A2. Post-flight average cells sampled, average aberration counts per subject, by postflight session.**

| Post-Flight Samples | | | |
| --- | --- | --- | --- |
| Post-flight Session | Average Aberration Count | Average # Cells Sampled per Crew-Mission | # Crew-Missions |
| First | 44.7 | 8909.8 | 39 |
| Second | 36.7 | 7595.6 | 31 |

**Note: Although only 5 of 38 astronauts participated in two missions, 29 astronauts had aberration data for a second post-flight session within a mission.** **Two of these 29 astronauts participated in two missions and had second-session data for both of their Stwo missions making a total of 31 crew-missions with at least two post-flight sessions.**

Figure A1. Predicted baseline CAR = ($exp(\beta_{0}+W_{0i})$ for three age groups (age < 45 yrs, 45 < age < 50 yrs, and age ≥ 50 yrs). Ages are grouped to preserve protection of privacy.

Figure A2. Baseline average slope (Eq 3) for three age groups (age < 45 yrs, 45 < age < 50 yrs, and age ≥ 50 yrs). Ages are grouped to preserve protection of privacy.

**PART TWO –Poisson Regression Analyses run with Stata Software**

- Pre-Flight model with random intercepts (W0) and random slopes (W1)

(for purposes of post-flight prediction)

Note: “x” = dose (Gy); age50 = Age-50 yrs; ifem = 1 if female, = 0 if male; post = 0 signifies pre-flight. “isub” is the subject number.

“n” is the number of cells sampled and “nab” is the number of total aberrations in each observation. There are 172 pre-flight observations contributing to the pre-flight dose-response model.

Note that this analysis is run with x > 0 (highlighted below) so that the baseline observations are withheld for the postflight analysis.

**. mepoisson t x age50 ifem if post==0 & x <=.55 & x > 0,exposure(n) ||isub:x,cov(uns) nolog**

**Mixed-effects Poisson regression Number of obs = 172**

**Group variable: isub Number of groups = 43**

**Obs per group:**

**min = 4**

**avg = 4.0**

**max = 4**

**Integration method: mvaghermite Integration pts. = 7**

**Wald chi2(3) = 278.98**

**Log likelihood = -539.44597 Prob > chi2 = 0.0000**

**------------------------------------------------------------------------------**

**t | Coef. Std. Err. z P>|z| [95% Conf. Interval]**

**-------------+----------------------------------------------------------------**

**x | 3.666809 .2223253 16.49 0.000 3.231059 4.102558**

**age50 | .0307235 .0118998 2.58 0.010 .0074003 .0540466**

**ifem | .1808011 .1153836 1.57 0.117 -.0453465 .4069488**

**_cons | -5.557825 .0999618 -55.60 0.000 -5.753747 -5.361904**

**ln(n) | 1 (exposure)**

**-------------+----------------------------------------------------------------**

**isub |**

**var(x)| 1.013879 .4586969 .4177217 2.460848**

**var(_cons)| .2734366 .0835362 .1502487 .4976256**

**-------------+----------------------------------------------------------------**

**isub |**

**cov(x,_cons)| -.4649322 .1839583 -2.53 0.011 -.8254838 -.1043806**

**------------------------------------------------------------------------------**

**LR test vs. Poisson model: chi2(3) = 207.05 Prob > chi2 = 0.0000**

Obtain random effects w0i and w1i

**predict W1 W0 if e(sample),reffects**

- Post-Flight Model: random intercepts(A0) [but not enough dose points to estimate random slopes].

Note: x0 = 0 means include only the pre-flight baseline observations (x = 0) and all postflight observations in the analysis. In this model “W1x” = $w_{1i}\times x_{ij}$.

**. mepoisson nab x age50 ifem W0 W1x if x0==0 ,exposure(n) ||isub: , nolog**

**Mixed-effects Poisson regression Number of obs = 116**

**Group variable: isub Number of groups = 43**

**Obs per group:**

**min = 1**

**avg = 2.7**

**max = 4**

**Integration method: mvaghermite Integration pts. = 7**

**Wald chi2(5) = 213.10**

**Log likelihood = -440.97656 Prob > chi2 = 0.0000**

**------------------------------------------------------------------------------**

**t | Coef. Std. Err. z P>|z| [95% Conf. Interval]**

**-------------+----------------------------------------------------------------**

**x | 14.74642 1.201595 12.27 0.000 12.39133 17.1015**

**age50 | .0247464 .0087547 2.83 0.005 .0075875 .0419053**

**ifem | .1616721 .0831806 1.94 0.052 -.0013588 .3247031**

**W0 | .7038476 .0866253 8.13 0.000 .5340652 .87363**

**W1x | 6.268415 1.611159 3.89 0.000 3.110601 9.426229**

**_cons | -5.751037 .0482957 -119.08 0.000 -5.845695 -5.656379**

**ln(n) | 1 (exposure)**

**-------------+----------------------------------------------------------------**

**isub |**

**var(_cons)| .0357084 .0108318 .0197045 .0647105**

**------------------------------------------------------------------------------**

**LR test vs. Poisson model: chibar2(01) = 66.73 Prob >= chibar2 = 0.0000**

- RBE Model

Note: In this model estimation , “x0E” = x if the observation is preflight; otherwise x0E = 0. Conversely, “x1E” -= x if the observations is postflight; otherwise x1E = 0.

**. mepoisson t x0E x1E age50 ifem,exposure(n) ||isub:x0E,cov(uns) nolog intpoints(21)**

**Mixed-effects Poisson regression Number of obs = 288**

**Group variable: isub Number of groups = 43**

**Obs per group:**

**min = 5**

**avg = 6.7**

**max = 8**

**Integration method: mvaghermite Integration pts. = 21**

**Wald chi2(4) = 830.69**

**Log likelihood = -1001.8181 Prob > chi2 = 0.0000**

**--------------------------------------------------------------------------------**

**t | Coef. Std. Err. z P>|z| [95% Conf. Interval]**

**---------------+----------------------------------------------------------------**

**x0E | 4.031142 .140433 28.71 0.000 3.755898 4.306385**

**x1E | 12.47462 1.058178 11.79 0.000 10.40063 14.54861**

**age50 | .0268452 .01132 2.37 0.018 .0046584 .049032**

**ifem | .1526223 .1096175 1.39 0.164 -.062224 .3674685**

**_cons | -5.678747 .0635761 -89.32 0.000 -5.803354 -5.554141**

**ln(n) | 1 (exposure)**

**---------------+----------------------------------------------------------------**

**isub |**

**var(x0E)| .4620095 .1596435 .2347066 .909445**

**var(_cons)| .102165 .0245894 .0637429 .1637467**

**---------------+----------------------------------------------------------------**

**isub |**

**cov(x0E,_cons)| -.113445 .0499523 -2.27 0.023 -.2113498 -.0155403**

**--------------------------------------------------------------------------------**

**LR test vs. Poisson model: chi2(3) = 515.13 Prob > chi2 = 0.0000**

**Note: The RBE estimate for an average subject would be the coefficient of X1E divided by the coefficient of x0E (12.47/4.03).**

**PART Three – Other Poisson regression analyses mentioned in passing, but not specifically detailed in main manuscript**

- Post-Flight Model with dose rate (xod) instead of dose (x)

**. mepoisson t xod age50 ifem W0 W1xod if x0==0 ,exposure(n) ||isub: , nolog**

**Mixed-effects Poisson regression Number of obs = 116**

**Group variable: isub Number of groups = 43**

**Obs per group:**

**min = 1**

**avg = 2.7**

**max = 4**

**Integration method: mvaghermite Integration pts. = 7**

**Wald chi2(5) = 221.57**

**Log likelihood = -434.99746 Prob > chi2 = 0.0000**

**------------------------------------------------------------------------------**

**t | Coef. Std. Err. z P>|z| [95% Conf. Interval]**

**-------------+----------------------------------------------------------------**

**xod | 2611.432 203.9626 12.80 0.000 2211.673 3011.191**

**age50 | .0262092 .0088944 2.95 0.003 .0087765 .0436419**

**ifem | .1498397 .0846426 1.77 0.077 -.0160568 .3157363**

**W0 | .7078997 .0882692 8.02 0.000 .5348952 .8809043**

**W1xod | 1266.156 278.9218 4.54 0.000 719.4791 1812.832**

**_cons | -5.761603 .0489466 -117.71 0.000 -5.857537 -5.665669**

**ln(n) | 1 (exposure)**

**-------------+----------------------------------------------------------------**

**isub |**

**var(_cons)| .0372768 .0110457 .0208552 .066629**

**------------------------------------------------------------------------------**

**LR test vs. Poisson model: chibar2(01) = 74.83 Prob >= chibar2 = 0.0000**

**Note this model fits the data slightly better than the one for dose (log likelihood = ‑435.00 vs ‑440.98 for the dose-based model), but the overall message is the same.**

- Post-Flight Model with stable translocations instead of total aberrations

Note: Here, “nab” refers to the number of translocations

**. mepoisson nab x age50 ifem W0 W1x if x0==0 ,exposure(n) ||isub: , nolog**

**Mixed-effects Poisson regression Number of obs = 116**

**Group variable: isub Number of groups = 43**

**Obs per group:**

**min = 1**

**avg = 2.7**

**max = 4**

**Integration method: mvaghermite Integration pts. = 7**

**Wald chi2(5) = 194.27**

**Log likelihood = -397.99553 Prob > chi2 = 0.0000**

**------------------------------------------------------------------------------**

**nab | Coef. Std. Err. z P>|z| [95% Conf. Interval]**

**-------------+----------------------------------------------------------------**

**x | 15.28648 1.369587 11.16 0.000 12.60214 17.97082**

**age50 | .0280734 .0081234 3.46 0.001 .0121519 .0439949**

**ifem | .1695448 .0775072 2.19 0.029 .0176335 .3214562**

**W0 | .7506314 .0989755 7.58 0.000 .556643 .9446198**

**W1x | 4.182018 2.035283 2.05 0.040 .1929354 8.1711**

**_cons | -6.038841 .0472489 -127.81 0.000 -6.131447 -5.946235**

**ln(n) | 1 (exposure)**

**-------------+----------------------------------------------------------------**

**isub |**

**var(_cons)| .0249269 .0089557 .0123268 .0504065**

**------------------------------------------------------------------------------**

**LR test vs. Poisson model: chibar2(01) = 30.51 Prob >= chibar2 = 0.0000**

**Note: This model fit is similar to the postflight model for total aberrations, but the standard errors tend to be higher, reflecting the smaller number of damaged cells. In particular the mean baseline translocation rate is estimated to be exp(-6.0388) = 0.0024 as compared with exp(-5.751) = 0.0032 for total aberrations.**
